# The role of oral health literacy in shaping health behaviors among migrants in Norway. An integrative review

**DOI:** 10.1186/s12903-025-07097-6

**Published:** 2025-11-10

**Authors:** Dixie Brea Larios

**Affiliations:** Research Department at the Oral Health Center of Expertise in Western Norway, Vestland Council County, Postboks 2354 Møllendal, Bergen, 5867 Norway

**Keywords:** Oral health literacy, Health behavior, Acculturation, Migrant health

## Abstract

The escalating disparities in oral health represent a pressing global health concern, particularly in the migrant population. Poor oral health and limited oral health literacy (OHL) can lead to enduring health complications such as tooth decay and obesity, significantly diminishing quality of life. Despite its crucial role in overall well-being, oral health is often disregarded during acculturation. Furthermore, individuals with a migration background may face increased risk factors for poor oral health. Identifying the barriers to optimizing the overall health of migrant populations is necessary to address this neglect. Intervention programs have emerged as a beacon of hope for reducing oral health inequalities, enhancing knowledge, and fostering preventive practices. Additionally, there are currently no validated tools available for assessing oral health literacy (OHL) in migrant populations in Norway. A thorough review of the literature is needed to address this gap. An in-depth analysis of the literature and available tools for OHL assessments in clinical settings and primary care is presented in this integrative review, emphasizing their advantages and limitations. Implementing the PRISMA model, this integrative review was based on a systematic search of five electronic databases (PubMed, PsycINFO, EBSCO, Web of Science, and Google Scholar) during the winter and summer of 2024. Of the 408 articles identified, twenty-five studies, including six dental studies, addressed self-reported health, attitudes, inequalities, and system navigation influencing oral health literacy and health behavioral aspects among migrants in Norway. Methodological quality, content analysis, and inclusion criteria focused on oral health outcomes, societal factors, cultural differences, and beliefs were assessed thematically for the selected studies. Results from studies indicated positive associations between OHL and risk factors, barriers, health perceptions, and healthcare experiences within the limited studies related to OHL, particularly health inequalities and health behavior themes. Further research is needed to identify the impact of interventions, oral health literacy, and behavioral changes on migrant health during acculturation. Longer-term studies and tools for improving oral health literacy should be recommended to ensure validity and a more comprehensive understanding of the applicability and effectiveness within the Norwegian population. Improving migrants’ health outcomes requires highlighting the importance of health behavior during the acculturation process.

**Supplementary Information:**

The online version contains supplementary material available at 10.1186/s12903-025-07097-6.

## Background

Oral diseases such as tooth decay and periodontitis are linked to cardiovascular diseases, obesity, and diabetes, underscoring the significant impact of oral health literacy on overall health [1]. Poor oral health can negatively impact a person’s quality of life and psychological well-being, incur socioeconomic costs, and pose ongoing challenges, such as limited access to healthcare services and low health literacy. When migrant health is significantly influenced by inequality and institutional constraints, rather than the conditions in the countries of origin, these factors can hinder the migration process [1]. Systematic reviews (e.g., 1, 2, 3) have stated a gap in oral health literacy among migrant populations and ethnic minorities, with issues often arising from societal influences, cultural traditions, language barriers, and limited access to health information. Not only do the experiences of stressful situations and dealing with unhealthy lifestyles and dietary habits affect migrant health [2–4], but health behaviors are also shaped by the knowledge of healthcare systems and financial situations [5, 6], directly resulting in poor health outcomes and limited health literacy. The lack of validated tools to measure oral health literacy in migration research highlights an important gap in the literature and the limits in providing valuable insights into migrant health challenges and tailoring interventions to improve their health outcomes.

Health literacy can strongly predict individuals’ health status, behaviors, and outcomes, influencing various health indicators, such as healthcare access, mental health, and oral health-related quality of life [7]. Health inequalities can be attributed to several factors, such as the impact of the *healthy immigrant effect* and policy variations from the home country and new environments [8–12]. Health literacy and cultural background play an essential role in understanding how and why social determinants of health influence migrant health. Information about health literacy status in, for example, European countries has remained scarce [13], including oral health literacy. The prevailing belief that health outcomes are a central difference between migrant and host populations includes more evidence for long-term effects [11, 14–17]. As migrant mobility continues, behaviors and cultural aspects are carried into different settings, resulting in a more sustainable global focus on the impact of poor oral health and oral health outcomes.

### Oral health literacy

Oral health promotion initiatives (e.g., [90]) have helped define oral health literacy as the “degree to which individuals have the capacity to obtain, process, and understand basic oral health information and services needed to make appropriate health decisions” [18]. Limited oral health literacy has been linked to poor oral health outcomes in both migrant and non-migrant populations [1]. However, research has indicated that non-migrants may generally have higher oral health literacy than those with a migration background [5, 19]. In Scandinavian countries such as Sweden, Norway, and Denmark, migrants have encountered difficulties in accessing preventive and regular dental care; often dependent on emergency rooms, presenting higher caries incidence among children of migrant parents compared to children of Scandinavian parents [11, 12, 20, 21]. Educational and intervention programs have been developed to address these inequalities in oral health, especially among children from migrant families [22]. In Norway, a significant proportion of the population has faced challenges in understanding health information. According to reports by Le and colleagues [23], nearly 20% of the Norwegian population scored at the lowest level of health literacy (below level 1), including individuals from migrant populations [24]. Furthermore, digital health literacy, encompassing access to digital health information and overall digital skills, is influenced by factors such as age, gender, and educational background. Individuals aged over 65, particularly those with long-term illness, often report very low digital skills [23]. These findings suggest that many individuals in Norway may not be digitally prepared or equipped to seek out essential health services, whether for oral health care or specific health information, when confronted with a health problem. As a result, socio-economic implications and increased health costs may arise. Additionally, individuals from migrant backgrounds who have limited proficiency in Norwegian may be underrepresented in health data. Filling the gap in oral health literacy among migrants in Norway while keeping the needs of the population in mind to improve health services is essential.

The global focus on oral health literacy has increased to improve the oral health status of the population, serving as a good predictor of outcomes beyond educational levels. Previous research has explored various models and frameworks to understand how OHL influences oral health knowledge, behaviors, and outcomes [25, 26]. The oral health literacy measurement tools often used are the Rapid Estimate of Adult Literacy in Dentistry (REALD), developed from REALM [27], and the Test of Functional Health Literacy in Dentistry (ToFHLiD), developed from ToFHLA [28]. Other OHL tools have been developed in recent years, such as the Health Literacy in Dentistry scale (HELD) [29] and the Oral Health Literacy instrument (OHLi) [30].

Few studies have been conducted in Norway with the attempt to measure oral health literacy by developing and validating instrument tools, such as the Adult Health Literacy Instrument for Dentistry (AHLID) [31–33]. However, non-existent studies in migration research make it difficult to identify strengths and weaknesses in OHL. This gap highlights the urgent need to further investigate the oral health literacy of migrant populations. Understanding this aspect is crucial for targeted interventions to improve oral health outcomes and reduce disparities in dental care access and quality in the Norwegian society. Therefore, exploring existing literature from an inclusive perspective to provide a synthesis of knowledge and significant research findings is important. A more comprehensive approach is needed to ensure the well-being of both migrants and host communities, ultimately to improve health outcomes, including oral health literacy, health behaviors, and health-related effects.

### Comprehensive intervention studies

Current review updates have examined whether acculturation factors are linked to specific diseases among migrants, influencing their lifestyles and dietary habits [1, 34]. Having a migration background can increase the risk of poor oral health, and migrant populations are widely reported to suffer from poor oral health, highlighting the gaps in oral health literature, such as differences in caries restoration and plaque index, negative oral health beliefs, and lower dental service utilization [1, 35]. Additionally, health knowledge is a crucial factor in determining engagement in health-related behaviors, as well as the interaction pathways from the benefits and drawbacks of those behaviors [26, 36, 37]. Theoretical models such as the COM-B model of behavior [38] and the Information-Motivation-Behavioral skills model (IBM) [26], have been used for behavioral change interventions. Moreover, frameworks such as the theory of motivation, encompassing Maslow’s [39] hierarchy of needs and Self-Determination Theory (SDT) [40], as well as the Theory of Planned Behavior [41], have also been used to collect information regarding health knowledge and intention in the migrant populations linking beliefs and behaviors [41, 42]. These intervention models and theoretical frameworks can provide a structured approach to understanding the factors influencing health behavior in research studies. This understanding can help design effective interventions that promote successful behavior modifications, particularly in Oral Health Literacy (OHL) in diverse populations, such as migrants. Utilizing both theoretical models of behavior and OHL measures is essential for comprehensive interventions to address the challenges faced by migrant populations and improve their overall well-being and oral health outcomes. A preliminary scoping of the literature aims to consolidate existing research on the role of oral health literacy (OHL) in shaping health behavior among migrant groups in Norway. This integrative review aims to explore the effectiveness, benefits, and challenges of the role of OHL in the migrant population by encompassing diverse studies.

Systematic reviews (e.g., 1, 21) have indicated that few studies relate to OHL in migrant communities and its influence on oral health behavior in Norway. To evaluate current evidence and describe oral health literacy and health behavioral changes in the process of migration, a review of the oral health literature is crucial. This integrative review seeks to summarize past experimental and theoretical data to develop a broad understanding of concepts and issues related to the intersections of oral health literacy and migration in Norway. Synthesizing studies on factors associated with oral health literacy influencing and promoting caries prevention, dietary habits, and health behavioral aspects is the aim of this integrative review.

## Method

### Eligibility criteria

This integrative review was undertaken to gather information and synthesize data from different viewpoints and research methods studies [43]. The existing body of literature on oral health literacy in migrants is diverse, encompassing theoretical concepts and methodological approaches related to oral health research and the migration process. While also aiming at the quality of research assessment [44, 45], this integrative review prioritizes identifying a broader range of the literature, providing critique by addressing emerging topics, and exploring the relationships between oral health literacy and health behavior [43, 46]. This integrative review has been registered on the Open Science Framework (OSF) under the CC-By Attribution 4.0 International license.

The PICOS framework involved migrants of all ages (e.g., children, adolescents, and adults), concerning the topic of oral health literacy, acculturation aspects, and health behavioral changes indicating health outcomes. The research questions “What indicators of oral health literacy are being explored among migrant populations in Norway?” and “How is the concept of oral health literacy associated with health behaviors and health outcomes?” guided the search strategy.

- P: Migrant population in Norway – all ages.
- I: Exploration of indicators and assessment of Oral Health Literacy.
- C: Comparison between different levels of oral health literacy for the assessment.
- O: Health behaviors and health outcomes.
- S: Observational studies, cross-sectional studies, qualitative studies, surveys.

The inclusion criteria incorporated peer-reviewed primary research, and both qualitative and quantitative studies conducted in Norway, with a variety of data collection methods and study designs. The review relied on studies published in English, including open-access publications, released within the past 20 years. Dissertations, editorials, reviews, and secondary sources (e.g., grey literature and non-English reports) were excluded. Studies written in the Norwegian language and published in Norwegian research journals were not considered to maintain consistency and quality control. This approach also addressed resource constraints, accessibility, and relevance, facilitating the interpretation and extraction of data from the selected studies [47, 48].

### Search strategy

This integrative review followed the PRISMA guidelines for reporting systematic reviews [49]. A research strategy was developed to explore aspects related to oral health literacy and migration by incorporating various sources and methodologies. This strategy generated new insights to summarize the existing literature on oral health behaviors. To ensure quality and rigor, a five-stage framework was provided for the methodological strategy [43, 50]. See Table 1 for an overview of the various stages in this integrative review.

**Table 1:** Stages of the integrative review following Toronto & Remington’s framework [36]

Stage	Studies
Problem identification	Migrants with low oral health literacy face multiple challenges related to their oral health, health behaviors, and overall well-being. This integrative review aims to explore the role of oral health literacy within the migrant population and its connection to health behaviors in Norway. It will examine how this concept is perceived and identify the indicators being studied.
Literature search	A comprehensive search strategy was conducted using five search databases, including PubMed, PsycINFO, EBSC, Web of Science, and Google Scholar. The search terms focused on oral health literacy, migration, and health behavior, along with clearly defined inclusion and exclusion criteria. This process resulted in 270 potentially relevant studies, which were screened for eligibility. A final assessment of twenty-five reports was selected, including 6 dental studies, comprising 1 mixed methods study, 18 quantitative and 6 qualitative studies.
Data evaluation	Critical appraisal skill programs such as CASP and the Mixed Methods Appraisal Tool were used to meet the gold standard criteria for the evaluation of studies during the appraisal and reference stages (1, 2).
Data analysis	Covidence and End-Note programs were used to extract information about the research questions and the study results relevant to the aims of the integrative review. NVivo program was used to code themes for the conceptual map of outcomes and key themes on OHL. The checklists supported the quality of the data extraction (program references).
Presentation of findings	A flow chart was created for the review process. The coding frame and cluster analysis integrated two main themes: Health literacy and health inequalities (see figure 2). The research questions focused on oral health literacy and health behavior among migrants. Results were summarized in a table (see table 2).

A comprehensive search strategy was implemented using specific terms related to oral health literacy in the context of migration and health behavior. This review focused on studies addressing oral health literacy in migrant groups of all ages, covering the period between January 2004 and September 2024. The search was conducted between the winter and summer of 2024 across various databases, including PubMed, PsycINFO, EBSC, Web of Science, and Google Scholar. Furthermore, search modifications were made to include other relevant sources such as manual searches of both peer-reviewed and grey literature. Boolean connectors (AND, OR, and NOT) were employed to combine search terms and keywords such as “oral health literacy,” “health literacy”, “migrants and immigrants”, “health behavior”, and “acculturation or cultural differences” were added to the search strategy. See Additional file 2 in the Supplementary material for complete search strings.

### Data collection process

**Fig. 1:**
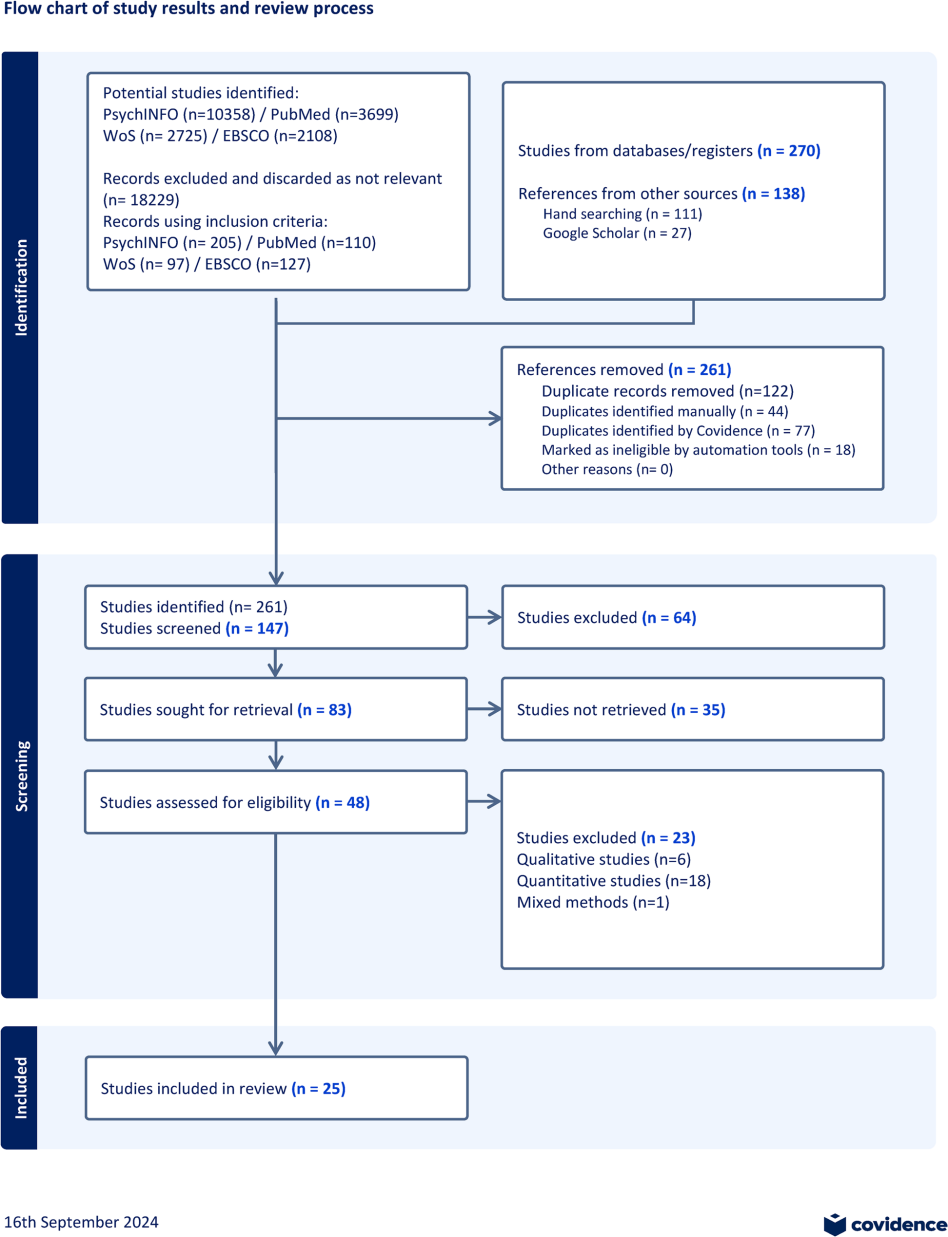
Flow chart of study results and review process

Data was collected on study design, sample size, type of intervention, and key findings. The search terms were based on their appearance in abstracts and keywords. The primary factors influencing oral health literacy include caries-related interventions, the understanding of health information, self-reported health, attitudes, health outcomes, dietary habits, health behaviors, and cultural differences. All included studies were reviewed, critically appraised, and carefully selected with the necessary tools following the inclusion criteria and main research question. Each database was recorded, and studies were exported to the EndNote [60] software program, which helped with hand searching, reference management, and extraction of studies relevant to the topic and research questions. The Covidence program [62] was a useful tool to streamline the review process, effective screening, and data management. The screening included all titles and abstracts for the inclusion criteria and full-text articles were reviewed to determine the final assessment. See Additional file 3 in the Supplementary material for excluded studies. Quality criteria instruments were used (i.e., critical appraisal skills checklist CASP) [66]. The Covidence flow chart included a comprehensive overview of the study selection process and final selection. See Fig. 1 for an overview of the selection process in a PRISMA flow chart.

### Thematic analysis

**Fig. 2:**
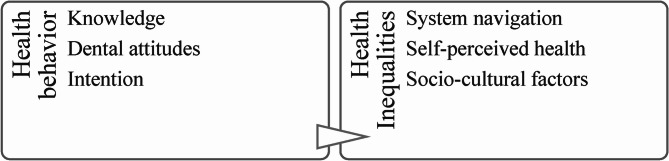
Oral health literacy themes. Coding framework and cluster analysis of the relationship of the study outcomes and key themes

Thematic analysis was conducted using the NVivo [61] software program to familiarize with the content, manage data segments, capture main ideas, and identify patterns [50, 69]. The coding frame and cluster analysis of themes resulted in two main themes: Health behavior and health inequalities, identified from the selected studies. The themes encompassed conceptual associations of oral health literacy with health behavior such as knowledge, attitudes, and intentions regarding healthy habits, understanding of the health system, and socio-cultural factors. Figure 2 presents an overview of the main identified themes.

### Data items

Oral health literacy played the role of a mediator through various factors such as knowledge, socioeconomic factors (e.g., education and employment), and adapted health behaviors. In addition, other intervention characteristics (e.g., values and needs), and the influence of acculturation aspects. Consequently, the search strategy identified 408 articles that were related to oral health literacy (See Fig. 1). After the deletion of duplicates, 147 articles remained. Eighty-three potential papers were identified as potentially relevant after the initial screening for titles and abstracts. Full text was obtained from forty-eight studies to assess the eligibility. Each of the studies was appraised following the inclusion and exclusion criteria, revised, and subsequently excluded after eliminating irrelevant studies, leaving twenty-five studies in the final review.

Data from the twenty-five papers were summarized, thematically analyzed and systematically synthesized to identify the relationships between oral health literacy and health behavior. Consistent with the final stages of Toronto & Remington’s [43] framework, an overview of the studies involving oral health literacy and health behavior among migrants was developed. The studies were charted by adding the author, publication year, sample size, methodology, and key findings (see Table 2).

**Table 2.** Overview of studies included in this review

Study	Authors	Year	Sample Size	Methodology	Key Findings
1. Public health challenges of immigrants in Norway: a research review. NAKMI report.	Abebe DS.	2010 [51]	222	Qualitative	Poor health conditions, multiple risk factors related to pre- and post-migration experiences, socioeconomic conditions, and individual backgrounds (e.g., Lifestyle and diet-related disorders, mental health problems, infectious disease, reproductive health). This report provides a more comprehensive understanding of the study findings offering different data and insights.
2. Renegotiating formal and informal care while aging abroad: Older Pakistani women’s healthcare access, preferences and expectations in Norway.	Arora S and colleagues	2020 [52]	23	Qualitative	Renegotiating expectations of informal care in light of the *nazaam* (or social system and practices) of Norway, fear of being left behind in residential care homes, disloyalty and shame of being cared for by outsiders, perceptions about the quality of formal care, and concerns about mixing with other cultures and genders.
3. Somali immigrant women’s knowledge of and experiences with folic acid supplement use before and during pregnancy: A qualitative study from Norway.	Aw-Nuur HA and colleagues	2024 [49]	150	Mixed Methods	Attitudes towards life and pregnancy on how health advice is received regarding the importance of folic acid. The healthcare system should tailor information to meet the needs of migrant women from Somalia; provided by someone they trust, in their first language, including visual aids for all women before their first pregnancy.
4. A controlled weight loss intervention study among women of Somali background in Norway	Bohler L and colleagues	2024 [53]	168	Interventional	Emphasis on lifestyle changes through self-empowerment and self-efficacy. Modest non-significant effect on weight change after 12 months. Longer-duration studies and childcare provision are needed.
5. Physical Activity Levels and Perceived Changes in the Context of Intra-EEA Migration: A Study on Italian Immigrants in Norway.	Calogiuri G, Rossi A, Terragni L.	2021 [54]	531	Quantitative	Most participants felt equally or more physically active than if they had been in Italy. However, some reported a negative impact on their activity levels. No significant differences were found in overall physical activity between Italians and Norwegians. Lower education, age, and less urbanized areas indicated a more negative impact and fewer activities than their counterparts. Acculturation, gender, and social gradient were associated with health behaviors.
6. Self-management of type 2 diabetes among Turkish immigrants in Norway: A focus group study.	Cokluk B and Tokovska M.	2023 [55]	13	Qualitative	Understanding the role and responsibility of health care staff in T2DM treatment, assessing education courses and information, and applying knowledge and motivation to adapt to life with T2DM were major themes. Self-management was related to cultural, religious, and socio-economic backgrounds and experiences.
7. How do immigrants use primary health care services? A register-based study in Norway. European Journal of Public Health. 2015;25[1]:72 − 8	Diaz E and colleagues	2015 [49]	388,881	Quantitative National population register	Fewer immigrants used general practitioners (GPs) compared to natives. Older immigrants visiting GPs less than younger ones. Those from high-income countries were less likely to use emergency services than natives, while a higher percentage of those from other countries did. Differences in health or access barriers, with morbidity burden and length of stay as key factors.
8. Prospective register-based study of the impact of immigration on educational inequalities in mortality in Norway.	Elstad JI, Overbye E, and Dahl E.	2015 [56]	135,251 (1993) 298,831 (2008)	Quantitative Register-based data	Lower mortality rates than natives, and highly educated immigrants had mortality rates similar to those of highly educated natives. The rise in educational inequality in mortality in Norway during the 1990 s and 2000 s was not caused by immigration. Immigration slightly lowered overall mortality and reduced the educational gradient in mortality.
9. Health literacy: the missing link in improving the health of Somali immigrant women in Oslo.	Gele AA and colleagues	2016[57]	302	Quantitative	The majority of the women could not access, understand, and act upon health information and services, hindering their health decision-making. Unemployment and lower social integration were independent predictors of inadequate health literacy in this group.
10. Oral health challenges in refugees from the Middle East and Africa: a comparative study.	Høyvik AC and colleagues	2019 [58]	132	Quantitative	Half of the refugees reported oral impacts on daily performances (OIDP) with lower scores and higher numbers of decayed teeth, particularly those from the Middle East. The oral health was generally poor, with added extensive challenges. However, most refugees had prerequisites for good dentition and were provided the necessary treatment.
11. Changes in food habits and motivation for healthy eating among Pakistani women living in Norway: results from the InnvaDiab-DEPLAN study.	Johansen KS and colleagues	2010 [59]	198	Interventional	Significant differences between intervention and control for sugar-rich drinks and rapeseed oil. Culturally adapted education has the potential to change intentions for a healthier diet.
12. Maternity care through the eyes of Southern European immigrant parents in Norway	Herrero-Arias R and colleagues	2022 [60]	15	Qualitative	The thematic analysis highlighted key insights into reproductive health services and patient-provider interactions, revealing gaps between patient expectations and health facility procedures, especially in check-ups and childbirth education. Maternity care in the host country preferred a less interventionist approach, influenced by healthcare providers’ cultural perspectives.
13. Health risks among long-term immigrants in Norway with poor Norwegian language proficiency	Kjøllesdal MKR, Gerwing J, and Indesth T	2023 [13]	3993	Quantitative	Immigrants were more likely to face negative health conditions than the general population, with risks varying based on duration of residence, Norwegian language proficiency, and education level. In analyses adjusted for age and sex, immigrants showed higher odds of most negative health conditions, excluding hypertension. Those with long residence in Norway and poor language skills were at the highest risk.
14. Self-reported health and associated factors among the immigrant populations in Norway.	Madar AA, Strand BH, & Meyer HE.	2022[61]	221	Quantitative	Health status was generally good or very good. Women had poorer self-reported health than men. Patterns of self-reported health were associated with employment status, diabetes, stress, and sleeping problems, as well as age, gender, and psychosocial conditions. The study suggested further research and implementing community-driven and cultural lifestyle intervention programs.
15. Changes in food habits among Pakistani immigrant women in Oslo, Norway.	Mellin-Olsen T. & Wandel M.	2005 [62]	21	Qualitative	Life in Norway led to changes in eating patterns and habits. The Koctürk model indicated dinner as the main meal of the day. The focus group interviews revealed multiple influencing factors (e.g., health aspects, children’s preferences, work schedules, traditional beliefs, season, and access to foods).
16. Attitudes toward brushing children’s teeth—A study among parents with immigrant status in Norway.	Mustafa M, Nasir EF, and Åstrøm AN.	2021 [63]	233	Intervention	Parents with non-Western backgrounds generally indicated positive attitudes, strong subjective norms, and a solid sense of control regarding their children’s tooth-brushing habits. Behavioral low indulgence reflected a high level of knowledge about oral hygiene. Participants had sufficient knowledge and intentions concerning tooth brushing with some negative attitudes that may adversely affect their children’s oral health. Cultural influences and established habits when developing oral health prevention policies are considered.
17. Experiences and perceptions of body weight among Turkish immigrant women in Norway	Rieger EY and colleagues	2021 [48]	15	Qualitative	Participants perceived Turkish women as being more overweight than Norwegian women. Collective preferences for weight loss had generational shifts with barriers regarding social norms, exercise, and eating habits during long winters. Concerns about the health impacts of being overweight and the desire to maintain cultural food practices were expressed. Perspectives on weight were influenced by the transition towards the ideals of thinness in Turkey. Self-image related to weight was also situated within the context of being immigrants in Norway.
18. Tracking of parents’ attitudes to their children’s oral health-related behavior–Oslo, Norway, 2002–04.	Skeie MS and colleagues	2010 [47]	31	Quantitative (longitudinal study)	Education and immigrant status were significantly associated with belonging to the attitudinal risk group. Norwegian parents’ dental attitudes were more positive in 2004 than in 2002. Culturally tailored programs of dental health education are encouraged for more positive oral health attitudes.
19. Caries increment in children aged 3–5 years in relation to parents’ dental attitudes: Oslo, Norway 2002 to 2004.	Skeie MS and colleagues	2008 [53]	354 (2002) 304 (2004)	Quantitative	Attitudes to diet and parental indulgence were more related to caries increment in early childhood among immigrant parents than non-immigrant parents.
20. Associations Between Immigration-Related User Factors and eHealth Activities for Self-Care: Case of First-Generation Immigrants from Pakistan in the Oslo Area, Norway.	Tatara N and colleagues	2019 [50]	176	Quantitative	Positive associations with Urdu literacy, information seeking by Web portals or email subscriptions, communication in social networks Norwegian language proficiency and decision-making. Immigration-related factors may confound associations between general user factors and e-health activities.
21. Self-Rated Health Among Italian Immigrants Living in Norway: A Cross-Sectional Study.	Terragni L and colleagues.	2022 [64]	321	Quantitative	Most participants felt their health would have remained unchanged if they had continued living in Italy, while 23% perceived a negative impact. The most significant factors influencing health included age, eating habits, and the number of years residing in Norway. These were followed by trust in others, educational level, and health literacy.
22. Migration as a turning point in food habits: the early phase of dietary acculturation among women from South Asian, African, and Middle Eastern Countries living in Norway	Terragni L. and colleagues	2014 [65]	21	Qualitative	Abrupt changes in food habits in the first period after migration. Unfamiliarity with food in shops, uncertainty about meal formats and preparations, and fear of eating food prohibited by religion. Restriction of food consumption to familiar and saved food items. Findings indicate that the first period after migration represents a specific phase in the process of dietary acculturation. Enhancing confidence in food and familiarity with the new food culture are recommended.
23. The role of education for current, former and never-smoking among non-western immigrants in Norway. Does the pattern fit the model of the cigarette epidemic?	Vedoy TF.	2013 [66]	4060	Quantitative Oslo Health and Oslo Immigrant Health Studies	The prevalence of smoking among men varied significantly by nationality but was higher among Turks compared to Norwegian men. Higher education led to lower smoking rates among male immigrant groups, except for Sri Lankans. For women, smoking was almost nonexistent among most migrant groups, except Turkish and Iranian women who were similar to Norwegian women’s smoking rates. Turkish and Iranian women with secondary education were more likely to smoke compared to those with primary education who were more likely to be never-smokers. There is a need for targeted interventions addressing smoking behaviors in population subgroups. The lower rates among the women reflected strong social norms against smoking.
24. Dental avoidance behaviour in parent and child as risk indicators for caries in 5-year‐old children.	Wigen TI, Skaret E, and Wang NJ	2009 [67]	523	Quantitative	Results indicated that children having one or more missed dental appointments, behavior management problems, dental anxiety, and parents avoiding dental care were associated with caries experience at the age of 5 years. Based on parents’ education and origin, those avoiding dental appointments and experiencing child behavior management problems were indicators for dental caries in 5-year-olds.
25. The influence of immigrant background and parental education on overweight and obesity in 8-year-old children in Norway.	Ovrebo B. and colleagues	2023 [68]	1283	Quantitative	Population-based Norwegian Childhood Growth study. Children with immigrant backgrounds had a higher prevalence of overweight and obesity compared to children with non-immigrant backgrounds. Differences varied by region of origin but not by parental education.

### Risk of bias assessment

The evaluation of data and methodological quality of the included studies was assessed using the Critical Appraisal Program (CASP) [66] and the Mixed Methods Appraisal Tool (MMAT) [59] to meet the gold standard criteria. The CASP checklist was employed to assess the relevance of each qualitative study selected, revealing robust methodologies, along with potential biases in some cases. The findings underscore the importance of critical appraisal in interpreting study results and practice implications. Additionally, the MMAT was used to evaluate quantitative and mixed methods studies related to specific issues and appropriate methodologies and assess the validity of the studies selected. The reviewer appraised each study to identify potential sources of bias. The findings from these studies were evaluated in a descriptive manner.

### Effect measures

The key findings revealed a significant absence of oral health literacy measures and assessment tools in the selected studies. Additionally, the research highlighted various health outcomes and behavioral changes, including lifestyle modifications, that emerged for example, from intervention studies. These insights emphasize the need for more comprehensive approaches to evaluate and examine oral health literacy and its impact on overall health behavior and acculturation.

### Synthesis methods

For better support and transparency, the OSF and Covidence platforms, along with tools such as the EndNote software program, were used to extract the information related to the research questions and study results for this integrative review. Records were identified through database searches, and the NVivo 15 program was used for the thematic analysis and to create a coding framework that organized themes for conceptual understanding and certainty assessment regarding Oral Health Literacy. The Risk of bias assessment tools and adherence to PRISMA guidelines ensured the quality of the data extraction [49]. See Additional File 1 for the complete PRISMA checklist.

Add additional File 1 here.

## Results

### Study selection

This integrative review identified studies related to oral health literacy since 2004. The results yielded studies with different study designs and migrant populations living in Norway. The selected studies included reported health literacy and integration aspects, oral health outcomes, and self-reported health, comprising 18 quantitative (within 6 dental studies, including 3 intervention and 1 mixed-method study), and 7 qualitative studies.

A total of 408 references were identified by searching 205 through PsychINFO, 110 through PubMed, 97 through Web of Science, and 127 through EBSCO. One hundred and thirty-eight through hand searching and other sources (e.g., Google Scholar). Figure 1 presents details of the identification, screening, and selection process. Duplicate references were removed, and eventually, 261 references remained. After reviewing the abstracts, 147 were included in the study, with 35 records excluded for failing the PICOS criteria or being non-relevant, resulting in 48 full-text studies for eligibility assessment. Following this assessment, 25 studies were included in this integrative review. An overview of the studies is reported in Table 2.

### Study characteristics

The present study focused exclusively on migrant participants; therefore, studies that examined only the Norwegian population were excluded, however, they were referenced for their relevance to oral health literacy (OHL). The characteristics and main outcomes of the 25 studies [21, 22, 25–38] are presented in Table 2. Overall, the studies evaluated showed a low risk of bias and sufficient detail providing enough information and explaining the methods used.

### Results of synthesis

Twenty-five (*n* = 25) studies reviewed oral health literacy and health behaviors (see Table 2). These studies focused mainly on health outcomes, health literacy, and behavioral changes in the migrant population living in Norway. This review explored the role of oral health literacy as the mediator impacted by knowledge, socioeconomic factors (e.g., education and employment), and health behaviors. The results highlighted different indicators for oral health literacy that were consistent with the main themes, even though most studies did not directly address oral health research.

Four studies including Skeie et al. [53], Rieger et al. [63], Diaz et al. [51], and Tatara et al. [52] showed a positive association with OHL regarding information, help-seeking, and acculturation aspects among migrant participants as well as changes in health behavior, education, and prerequisites for good dentition. Studies by Kjøllesdal et al. [16], Herrero-Arias et al. [55], and Mellin-Olsen et al. [65], among others, were associated with integration aspects, healthcare access, and perceptions of healthcare quality. The socio-cultural aspects of most studies were related to oral health literacy in terms of knowledge of food intake, dietary and smoking habits, access to health information due to language barriers, and lack of trust in the institutions [54, 56]. Socioeconomic factors such as education were linked to health inequalities, particularly regarding mortality rates and higher prevalence of overweight and obesity. Studies by Ovrebo et al. [57] and Elstad et al. [58], demonstrate that individuals with lower educational attainment are at a higher risk of these conditions. These findings emphasize the need to prioritize educational initiatives and health resources not only for oral health literacy but also for health literacy in general.

The interventional studies [53, 64, 67, 68, 70] selected (see Table 2), highlighted the importance of lifestyle changes and positive attitudes in migrants, which could improve caries control and patient adherence to treatment plans and OHL. These studies suggest a shift in health behavior, particularly among migrant women. Factors such as self-empowerment and self-efficacy were associated with successful weight loss control presented in Arora et al. [64] study. The significant differences in these studies from food habits and intentions toward healthier choices were factors linked to oral health literacy and health behavioral changes. Other indicators associated with oral health literacy were access to and understanding of health information and knowledge about decision-making, influenced by socioeconomic and cultural factors. Overall, these studies highlighted the importance of having health information knowledge, and the ability to access healthcare services.

The qualitative studies [55, 63, 65, 71–74] highlighted the significance of self-management in health interventions while placing less emphasis on implementing structured intervention programs. In contrast, the included quantitative studies [16, 51–54, 56–58, 68, 75–79] underscored the need for additional research to explore the barriers migrants face. Moreover, culturally tailored educational resources should be developed, and the duration of studies should be extended to ensure more effective outcomes. By focusing on these strategies, the effectiveness of health initiatives may improve, fostering meaningful connections with diverse communities and enhancing oral health literacy.

### Risk of bias assessments

The inclusion criteria used in the research studies conducted in Norway encompassed a variety of data collection methods and study designs. Overall, these migrant health studies have offered valuable insights into the health status of migrants in Norway while highlighting the challenges faced in this area of research. Following the MMAT assessment, the majority of the selected quantitative studies demonstrated low risks. Some studies, as reported by Abebe and colleagues [71], may have portrayed migrants as a homogeneous group, as defined in Statistics Norway [80], overlooking differences in ethnicity, traditions, religious backgrounds, socioeconomic conditions, and motivations for migration. Conversely, some studies acknowledged that the length of stay can influence the integration process. Although factors related to both pre-migration and post-migration periods were insufficiently addressed, they may lead to poorly adjusted health outcomes. However, these elements were not the primary focus of the research questions and indicated lower risks of bias. The sampling techniques used in most studies included random sampling, convenience sampling, and data obtained from register data.

## Discussion

The key findings in this integrative review highlight the significant impact of oral health literacy within the migrant population in Norway, with some evidence supporting health behavioral changes. The selected studies collected information using various methodologies and data from migrant participants, particularly in the urban areas and major cities such as Oslo.

The findings indicate a positive development in physical activity and food consumption contributing to healthier behaviors despite the challenges migrants face in navigating an unfamiliar system. The review analysis also revealed that lower levels of education and limited language skills still hinder the health outcomes of migrants compared to the Norwegian population. While having a migration background does not directly cause health issues, the various associated risk factors place migrants at a disadvantage. These findings are consistent with previous research that identified the negative impact of health literacy on migrant health, such as in oral health beliefs, behavior, and levels of oral health knowledge [1].

Most of the study designs in the selected studies involved surveys, standardized questionnaires, interviews, and measurements of health literacy. Positive changes in dietary habits, oral health attitudes, and knowledge (e.g., patterns of self-reported health and no dental caries) were favorable among the migrant participants, including children with migrant backgrounds. These findings can be connected to studies focused on parents with young children and maternal oral health resources, resulting in the development of integrated models to maximize the existing primary health care system, and future training and educational modules aimed at primary care providers [81–84]. Recognizing the health needs of migrants is crucial by looking at the root causes of disease as well as addressing the social and economic determinants of health [5, 6]. This approach not only supports well-being but also promotes inclusiveness in health care access. By focusing on these positive findings, integrated models can lead to long-term improvements in oral health outcomes, bridging the gap in health disparities.

Oral health conceptual and theoretical models are less developed than those in the medical field. Even the emerging topic of commercial determinants of health (CDoH) requires further study, since the production and marketing of goods and services influence political and economic systems [85]. Comprehensive oral health interventions for migrants, including guidance provided in health stations, health monitoring, and follow-ups, may reduce caries levels, emphasizing lifestyle changes [64, 67, 70]. Despite these findings, future oral health interventions and further research are necessary to help reduce social inequalities and health disparities. Norwegian oral health literacy should be strengthened through programs that monitor and educate, not only migrants, but also the entire Norwegian population. This approach aims to raise awareness of potential health risks and promote healthier behaviors.

### Health behavior and health inequalities

The thematic analysis revealed key themes related to oral health literacy across the selected studies. One of the main themes identified was *health behavior*, which encompassed aspects of knowledge, attitudes, and intentions from the evaluated studies. Another main theme was *health inequalities*, which comprised socio-cultural aspects, healthcare system navigation related to acculturation, and self-perception of health as seen in the works of Calogiuri et al. [75], Johansen et al. [67], and Cokluk et al. [73]. The majority of the studies reviewed showed positive outcomes linked to oral health literacy indicators, particularly in terms of length of stay, which positively influenced the integration process. However, these indicators also revealed that some factors of acculturation could have a negative impact on oral health literacy regarding traditional differences, experiences, and perceptions of health care services. The thematic analysis highlighted that health inequalities may intersect with health behavior, mainly with key themes of socio-cultural factors influencing individuals’ dental attitudes and intentions towards oral health practices. The studies underscored the importance of culturally sensitive interventions to address these disparities and improve oral health literacy across diverse populations. Gaining insight into the relationship between these themes, more effective strategies can be developed to improve oral health outcomes.

Cognitive and social skills played a key role in oral health literacy, such as in the works of Bohler et al. [64], Mustafa et al. [70], Herrero-Arias et al. [55], and Mellin-Olsen et al. [65] and Vedoy et al. [54], influencing individuals’ motivation and intention, and their ability to access, understand, and use information that promotes and supports overall health [5, 86, 87]. These skills can relate to health inequalities, particularly regarding the differences in system navigation and health behavioral changes within the Norwegian population, which can also be associated with theoretical frameworks of health-related behaviors used in comprehensive intervention studies [26, 36–38]. As Skeie and colleagues [53] and Mustafa and colleagues [70] noted, barriers to accessing preventive healthcare and receiving regular dental care persist for the migrant population, including dental attendance, language difficulties, and understanding health information [88, 89]. These challenges can impact migrants’ quality of life and the integration process, contributing to more disparities. While cognitive and social skills are essential in enhancing knowledge, accessing and comprehending health information is crucial. Language barriers or socioeconomic status may contradict the notion that these skills alone can bridge health inequalities. Tailored intervention strategies can help address these disparities to ensure equitable access to health resources and effective provision. Understanding migrant health requires a broader migration perspective. Unlike previous research on oral health literacy in the Norwegian population, this integrative review emphasizes the overall importance of oral health, providing knowledge and understanding of the oral health impact on migrant health.

### Implications

Despite the high response rates and sample sizes from the studies selected, considering validated questionnaires for cross-cultural applicability may avoid potential information bias due to variances in health perceptions, attitudes, and language barriers. Studies with a mixed-methods approach or a prospective, longitudinal design to compare migrants with the Norwegian population should be considered. Developing and validating an Oral Health Literacy tool specifically designed for both migrants and the Norwegian population is crucial for accurately measuring and understanding health disparities. This tool would enable identifying needs and barriers encountered by various groups, facilitating effective interventions and changing behaviors over time. Offering valuable data on oral health literacy levels across diverse communities would enhance public health initiatives.

The majority of the studies have recommended further research within this population group. The challenges within the study population may lead to misleading conclusions, potentially undermining the validity of the study findings. Informed opinions and findings may suggest healthcare strategies that prioritize public health outcomes to mitigate issues of inadequate health literacy, including oral health literacy. Engaging in educational tools to accommodate various learning styles is essential for improving health behaviors.

The existing studies have highlighted the impact of migrant health on disease awareness and health management, and help-seeking behaviors may extend beyond dental visits and emergency rooms, also including the need for medical and mental health support. Some studies also considered the length of residence as an indicator, which may impact health behavior and integration aspects in Norway. These key findings can be the background for improvement. Oral health educational programs that are culturally sensitive and tailored to the needs of migrant populations are still essential. Community initiatives in familiar settings and integrating models within primary healthcare are necessary. Additionally, stricter regulation implementation and addressing alternative healthcare needs can benefit health behavior. Other recommendations can involve digital health, which is important for follow-ups, remote consultations, and mobile applications to provide easier ways to understand health information. Often, these aspects can be unreliable and challenging for health professionals and migrant groups; however, they may improve oral health literacy and the effectiveness of health services for better oral health outcomes. Social media platforms should work together with policymakers to ensure the integrity of information, protect users, influence health behaviors, and promote oral health literacy effectively.

### Limitations

The studies selected in this integrative review sought to address the research questions. Nevertheless, no studies conducted in Norway directly measured oral health literacy within the migrant population, and the use of appropriate tools and instruments may limit the intention of the integrative review. Future research should focus on developing cross-cultural oral health literacy tools and using reliable OHL instruments for the Norwegian population. Many of the methodologies employed in these studies involved participants primarily residing in urban areas and major Norwegian cities, such as Oslo. The lack of data from migrants in smaller urban settings or rural regions may influence future health outcomes and behaviors. Consequently, the findings may not represent the experiences of migrants in rural areas. Future research should prioritize qualitative studies to highlight the exploration across diverse geographical contexts.

This review was designed and conducted as an integrative review, incorporating a PRISMA checklist element to enhance methodological rigor following a systematic evaluation. However, the inclusion criteria limited studies to those published solely in English, thereby excluding potentially relevant research conducted and reported in Norwegian. While this language restriction was implemented to ensure consistency and control in data extraction and analysis procedures [47, 48], it may have introduced some selection bias, limiting the review from fully capturing its scope. Although oral health literacy research among migrants in Norway is limited, including health reports published in Norwegian could have strengthened the overall findings within the study population. Therefore, including potential evidence in non-English publications and a call for multilingual reviews are considered.

Furthermore, the cross-sectional design of some of the selected studies may not allow for causal inferences. The excluded papers published in non-peer-reviewed journals should be acknowledged and incorporated into future research initiatives. Including these works may offer a more comprehensive perspective for systematic reviews and meta-analyses, ensuring that a broader range of studies is considered in the context of oral health literacy. This approach can reveal valuable insights and trends that might otherwise be missed. Additionally, longitudinal studies are still needed to establish a causal relationship between oral health literacy and health behaviors. However, issues such as low participation rates and participant attrition over time may hinder these efforts.

Addressing challenges such as social factors, barriers, patient engagement, and data security are essential for optimizing interventions, educational programs, and new policies. Oral health offers a promising approach to oral disease management to explore long-term outcomes and cost-effectiveness, particularly within the migrant population. Creating and validating tools to effectively assess oral health literacy in migrant populations is vital for future research. These tools can yield important insights into the unique challenges, enabling the development of targeted interventions to enhance health outcomes. Addressing this gap will greatly improve our understanding of healthcare access and education for these communities and the entire Norwegian population. This integrative review highlights the importance of oral health literacy in the migrant population in Norway, emphasizing its role in study implementation to improve oral health in the migrant populations.

## Conclusion

The selected studies were systematically screened and assessed for methodological quality, content analysis, and inclusion criteria. Findings from this review suggest that oral health outcomes show promise despite limited research in Norway and the challenges influenced by social determinants and cultural barriers. By focusing on these aspects, future reviews can build a more comprehensive understanding of oral health outcomes. Additionally, future research should explore health behaviors, theoretical frameworks, and measurement tools to improve oral health literacy by formulating specific research questions and effective communication strategies that combine quantitative surveys and qualitative insights. Oral health is a crucial aspect of overall health yet often overlooked during the migration process. Incorporating additional Norwegian publications in future reviews should be considered in the review process to enhance analysis and broaden perspectives. Longitudinal studies can also help assess the long-term impacts of oral health interventions on health outcomes in the migrant population. Exploring health behaviors can provide more detailed insights into overcoming the identified challenges, which could enhance the implementation of solutions for those with limited oral health literacy. Collaborative efforts between healthcare providers, policymakers, and community organizations are important to develop comprehensive interventions to improve oral health and well-being. Future policies can work to address the barriers in oral health care and factors related to the need for oral health literacy.

## Supplementary Information

Supplementary Material 1

Supplementary Material 2

Supplementary Material 3

## Data Availability

All data generated or analyzed during this study are included in this published article [and its supplementary information files].

## Abbreviations

AHLID: Adult Health Literacy Instrument for Dentistry
CASP: Critical Appraisal Skills Program
COM-B: Capability (C), Opportunity (O), and Motivation (M) model of Behavior
HeLD: Health Literacy Dentistry scale
IBM: Information-Behavioral Skills model
MMAT: Mixed Methods Appraisal Tool
OHL: Oral Health Literacy
OSF: Open Science Framework
PICOS: Population, Intervention, Comparison, Outcome, and Study Design Framework
PRISMA: Preferred Reporting Items for Systematic Reviews and Meta-Analyses
REALD: Rapid Estimate of Adult Literacy in Dentistry
REALM: Rapid Estimate of Adult Literacy in Medicine
ToFHLiD: Test of Functional Health Literacy in Dentistry
ToFHLA: Test of Functional Health Literacy in Adults
