# Supplementary material for: The role of oral health literacy in shaping health behaviors among migrants in Norway. An integrative review
Source: BMC Oral Health. 2025 Nov 10;25:1766. doi: 10.1186/s12903-025-07097-6 (PMC12599096; doi:10.1186/s12903-025-07097-6)
Supplement: Supplementary file 1 — Supplementary Material 1 [file 12903_2025_7097_MOESM1_ESM.docx]

**Additional file 2. Documentation on the literature search for: The role of oral health literacy in shaping health behaviors among migrants in Norway. An integrative review**

The following databases were searched:

| **Database** | **Numbers of retrieved references** |
| --- | --- |
| PsychINFO: | 205 |
| WoS: | 97 |
| CINAHL (EBSCO): | 127 |
| PuBMed: | 110 |
| Google scholar/Handsearching: | 138 |
| Total number of retrieved references: | 704 |
| Number of references after deduplication: | 261 |
| Studies excluded: | 64 |
| Studies assessed for eligibility: | 48 |

All searches were done between January 2024 and September 2024.

Last search September 4^th^ and September 10^th^, 2024, by Dixie Brea Larios, Postdoctoral research fellow, Research Department, Oral Health Centre of Expertise in Western Norway.

All studies identified through database searches* (n= 150,504)

PsychINFO (n = 64755), PubMed (n =4,811), WoS (n = 61,402), EBSCO (n = 2,036), and Google Scholar (n=17500). All search string results are added from each database with keywords, Boolean operators, and filters.^[[1]](#endnote-1)^

**Potential studies identified from Databases:**

PsychINFO (n = 10358), PubMed (n = 3,699), WoS (n = 2,725), EBSCO (n= 2108).

Records using the inclusion criteria: PsychINFO (n = 205), PubMed (n = 110), WoS (n = 97), EBSCO (n=127), Google Scholar (n=27), Hand searching (n=95). See Figure 1 in the manuscript for the PRISMA flow chart.

**Search string results from databases:**

___________________________________________________________________________

1. **Web of Science: Clarivate**

**_______________________________________________________________________**

**ALL=(migrant* OR immigrant AND oral health literacy OR Oral health knowledge AND oral health behavior AND Oral health coping or oral health self-management AND balanced diet AND Obesity AND Migrant children AND Parents of migrant children)**

Refined by: and **Immigration** (Should – Search within topic) and **Acculturation** (Should – Search within topic) and **Norway** (Should – Search within topic) and **Scandinavian Countries** (Should – Search within topic) and **Self-efficacy** (Should – Search within topic) and **1999** or **2000** or **2001** or **2002** or **2003** or **2004** or **2005** or **2006** or **2007** or **2008** or **2009** or **2010** or **2011** or **2012** or **2013** or **2014** or **2015** or **2016** or **2018** or **2017** or **2019** or **2020** or **2021** or **2022** or **2023** or **2024** (Publication Years) and **Article** (Document Types) and **Early Access** or **Proceeding Paper** or **Book Chapters** or **Retracted Publication** or **Data Paper** or **Publication With Expression Of Concern** (Exclude – DocumentTypes) and **Spanish** or **German** or **French** or **Russian** or **Portuguese** or **Italian** or **Turkish** or **Slovenian** or **Croatian** or **Dutch** or **Polish** or **Czech** or **Chinese** or **Norwegian** or **Slovak** or **Korean** or **Swedish** or **Estonian** or **Unspecified** or **Ukrainian** or **Lithuanian** or **Japanese** or **Catalan** or **Afrikaans** or **Hungarian** or **Bulgarian** or **Danish** or **Greek** or **Serbian** or **Icelandic** or **Malay** or **Indonesian** or **Basque** or **Galician** or **Georgian** or **Arabic** or **Malayalam** or **Welsh** (Exclude – Languages) and **15 Life On Land** or **13 Climate Action** or **14 Life Below Water** or **16 Peace And Justice Strong Institutions** or **09 Industry Innovation And Infrastructure** or **07 Affordable And Clean Energy** (Exclude – Sustainable Development Goals).

**(ALL=(Immigrants OR migrants AND oral health literacy AND health behavior AND acculturation OR cultural differences AND Norway)) AND (PY==("2025" OR "2023" OR "2024" OR "2022" OR "2021" OR "2020" OR "2019" OR "2018" OR "2017" OR "2016" OR "2015" OR "2014" OR "2013" OR "2012" OR "2011" OR "2010" OR "2009" OR "2008" OR "2007" OR "2006" OR "2005" OR "2004") AND LA==("ENGLISH") AND DT==("ARTICLE" OR "REVIEW")) and Article or Review Article(Document Types)**

**Web of Science Core Collection**

[**61,402**](https://eur03.safelinks.protection.outlook.com/?url=https%3A%2F%2Fwww.webofscience.com%2Fwos%2Fwoscc%2Fsummary%2F2e529ee3-0b96-4e75-bc15-30071fe5ab05-010746ddc2%2Frelevance%2F1&data=05%7C02%7CDixie.Janice.Brea.Larios%40vlfk.no%7Ca6cde25ad7794110422808ddae786dfb%7C5b14945b0f8740ddacf35e5e21e6eb36%7C0%7C0%7C638858554463512940%7CUnknown%7CTWFpbGZsb3d8eyJFbXB0eU1hcGkiOnRydWUsIlYiOiIwLjAuMDAwMCIsIlAiOiJXaW4zMiIsIkFOIjoiTWFpbCIsIldUIjoyfQ%3D%3D%7C0%7C%7C%7C&sdata=wjLFedMLKIa4w0yp8dmRr2L%2BAKE6YhTCcSoYUoLQDXM%3D&reserved=0)

**Editions: A&HCI , ESCI , SCI-EXPANDED , SSCI**

[**https://www.webofscience.com/wos/woscc/summary/595ed6b2-9254-4b19-abcf-90c0ab9b49e4-010746e276/relevance/1**](https://eur03.safelinks.protection.outlook.com/?url=https%3A%2F%2Fwww.webofscience.com%2Fwos%2Fwoscc%2Fsummary%2F595ed6b2-9254-4b19-abcf-90c0ab9b49e4-010746e276%2Frelevance%2F1&data=05%7C02%7CDixie.Janice.Brea.Larios%40vlfk.no%7Ca6cde25ad7794110422808ddae786dfb%7C5b14945b0f8740ddacf35e5e21e6eb36%7C0%7C0%7C638858554463543681%7CUnknown%7CTWFpbGZsb3d8eyJFbXB0eU1hcGkiOnRydWUsIlYiOiIwLjAuMDAwMCIsIlAiOiJXaW4zMiIsIkFOIjoiTWFpbCIsIldUIjoyfQ%3D%3D%7C0%7C%7C%7C&sdata=COjOIelyzaUftx%2Fjt6ljhSG5%2FfIplBECp%2F6kVkINI7M%3D&reserved=0)**(Last search September 10th, 2024, 1:42pm)**

1. **PsychINFO**

**Any Field**: migrants *OR* **Any Field**: immigrants *AND* **Any Field**: oral health literacy *AND* **Any Field**: health behavior *OR*

**Any Field**: health behavioral changes *AND* **Any Field**: Acculturation *OR* **Any Field**: cultural differences *AND*

**Any Field**: Norway *AND* **Population Group**: Human *OR* Female *OR* Male *AND* **APA Full-Text Only** *AND*

**Open Access** *AND* **Year**: 2004 *To* 2024 - APA PsycInfo, APA PsycArticles, APA PsycBooks, APA PsycExtra

**_________________________________________________________________________**

1. **PubMed (MEDLINE)**

This message contains search results from the National Center for Biotechnology Information [(NCBI)](https://eur03.safelinks.protection.outlook.com/?url=https%3A%2F%2Fwww.ncbi.nlm.nih.gov%2F&data=05%7C02%7CDixie.Janice.Brea.Larios%40vlfk.no%7C095cabde34ea4a8e4ce308dcd19b64a4%7C5b14945b0f8740ddacf35e5e21e6eb36%7C0%7C0%7C638615712620247078%7CUnknown%7CTWFpbGZsb3d8eyJWIjoiMC4wLjAwMDAiLCJQIjoiV2luMzIiLCJBTiI6Ik1haWwiLCJXVCI6Mn0%3D%7C0%7C%7C%7C&sdata=1eVr1fvSuGDJOUqTod%2BG4xUb6u0GeQPG8HdC0yf88FA%3D&reserved=0) at the U.S. National Library of Medicine [(NLM)](https://eur03.safelinks.protection.outlook.com/?url=https%3A%2F%2Fwww.nlm.nih.gov%2F&data=05%7C02%7CDixie.Janice.Brea.Larios%40vlfk.no%7C095cabde34ea4a8e4ce308dcd19b64a4%7C5b14945b0f8740ddacf35e5e21e6eb36%7C0%7C0%7C638615712620259216%7CUnknown%7CTWFpbGZsb3d8eyJWIjoiMC4wLjAwMDAiLCJQIjoiV2luMzIiLCJBTiI6Ik1haWwiLCJXVCI6Mn0%3D%7C0%7C%7C%7C&sdata=z3nXTuSTkgUbQwaWOcmrFgmkTnXp4RURqdoNwgqX36U%3D&reserved=0). Do not reply directly to this message

**Sent On:** Tue Sep 10 09:20:49 2024

**Search:** (((((((migrants) AND (oral health literacy)) AND (health behavior)) AND (acculturation)) OR (cultural differences)) AND (Norway))) Filters: Clinical Trial, Meta-Analysis, Randomized Controlled Trial, Review, Systematic Review, Humans, English, Female, Male, from 2004 - 2024

**From:**dixie.brea@uib.no

Database: PubMed (searched via MEDLINE on [date]) Search Strategy: #1 "systematic review"[Title/Abstract] OR "meta-analysis"[Title/Abstract] #2 "reproducibility"[MeSH Terms] OR "reproducibility"[Title/Abstract] #3 #1 AND #2 Filters: English language, humans, published 2010-2024 Results: 1,247 records

1. **EBSCO/CINAHL (Ovid) ALL=(migrant* OR immigrant AND oral health literacy OR Oral health knowledge AND oral health behavior AND Oral health coping or oral health self-management AND balanced diet AND Obesity AND Migrant children AND Parents of migrant children)**. All search string results are added from each database with keywords, Boolean operators, and filters (even though the original string in certain databases may have been removed from their string search or lost due to a database or online issues).

<https://exports.ebscohost.com/sdc/9b2d0692-67d2-45db-8b97-21fdadf3891a.zip> Date: September 4th, 2024

| **Database** | **Platform** | **Search String** | **Date Searched** | **Results** |
| --- | --- | --- | --- | --- |
| PubMed | MEDLINE | ("systematic review"[Title/Abstract] OR "meta-analysis"[Title/Abstract]) AND "reproducibility"[MeSH Terms] | 2024-01-15 | 1,247 |
| CINAHL/Embase | Ovid | exp systematic review/ OR meta-analysis.mp. AND reproducibility.mp. | 2024-01-15 | 892 |

1. Searches were conducted across the four databases and google scholar, to reduce the risk of missing key studies due to the any loss of a single search string. With time, original strings may have been removed or lost due to database or online issues. This limitation is transparaently reported in this document to identify any relevant or potential records that may have been missed after Sepetmber 10th, 2024. In addition to the database searches and ensure consistency, manual screening and reference checking were done with the EndNote and Covidence Software programs to create the PRISMA flow chart [↑](#endnote-ref-1)
