# Supplementary material for: The role of oral health literacy in shaping health behaviors among migrants in Norway. An integrative review
Source: BMC Oral Health. 2025 Nov 10;25:1766. doi: 10.1186/s12903-025-07097-6 (PMC12599096; doi:10.1186/s12903-025-07097-6)
Supplement: Supplementary file 2 — Supplementary Material 2 [file 12903_2025_7097_MOESM2_ESM.docx]

**PRISMA Excluded Studies Table**

| **Study ID** | **Author(s) & Year** | **Title** | **Journal/Source** | **Reason for Exclusion** |
| --- | --- | --- | --- | --- |
| #173 | Carteret (2013) | How culture affects oral health beliefs and behaviors | Dimensions of Culture | Wrong study design |
| #76 | Edberg (2007) | Essentials of health behavior: Social and behavioral theory in public health | Jones & Bartlett Publishers | Wrong study design |
| #170 | Elyasi et al. (2015) | Impact of sense of coherence on oral health behaviors: a systematic review | PLoS One | Wrong setting |
| #171 | Elyasi et al. (2020) | Modeling the Theory of Planned Behaviour to predict adherence to preventive dental visits in preschool children | PLoS One | Wrong setting |
| #77 | Hayden (2022) | Introduction to health behavior theory | Jones & Bartlett Learning | Wrong study design |
| #102 | Lumsden et al. (2021) | Protocol for a family-centered behavioral intervention to reduce early childhood caries: the MySmileBuddy program efficacy trial | BMC Oral Health | Wrong setting |
| #154 | Muller et al. (2024) | Impact of an Oral Health Education Program on the Oral Health Literacy of Refugees | Journal of Immigrant and Minority Health | Wrong setting |
| #103 | Novrinda & Han (2022) | Oral health inequality among Indonesian workers in South Korea: role of health insurance and discrimination factors | BMC Oral Health | Wrong setting |
| #133 | Ntoumanis et al. (2021) | A meta-analysis of self-determination theory-informed intervention studies in the health domain: effects on motivation, health behavior, physical, and psychological health | Health Psychology Review | Wrong study design |
| #157 | Ramos-Gomez & Kinsler (2022) | Addressing social determinants of oral health, structural racism and discrimination and intersectionality among immigrant and non-English speaking Hispanics in the United States | Journal of Public Health Dentistry | Wrong setting |
| #162 | R'Ong et al. (2021) | Oral Health Seeking Behaviour among Non-Malaysian Adults in Malaysia | Medical Journal of Malaysia | Wrong setting |
| #110 | Shi et al. (2021) | Application of the extended theory of planned behavior to understand Chinese students' intention to improve their oral health behaviors: a cross-sectional study | BMC Public Health | Wrong study design |
| #84 | Shmarina et al. (2024) | Oral health literacy among migrant mothers in Sweden. A qualitative study | Acta Odontologica Scandinavica | Wrong setting |
| #111 | Svendsen et al. (2020) | Associations of health literacy with socioeconomic position, health risk behavior, and health status: a large national population-based survey among Danish adults | BMC Public Health | Wrong setting |
| #120 | Tsakos et al. (2023) | Reflections on oral health inequalities: Theories, pathways and next steps for research priorities | Community Dentistry and Oral Epidemiology | Wrong study design |
| #95 | Wang et al. (2022) | Teenager dietary behavior and health literacy in China: influencing factors and coping strategies | Archives of Medical Science | Wrong setting |
| #82 | Wendt et al. (1996) | Analysis of caries-related factors in infants and toddlers living in Sweden | Acta Odontol Scand | Wrong setting |

**Summary of Exclusion Reasons**

- **Wrong study design**: 6 studies (35.3%)
- **Wrong setting**: 11 studies (64.7%)
- **Total excluded studies**: 17 from Covidence Screening.

**Notes**

- All exclusions were made on November 20, 2024
- Studies excluded for "Wrong study design" included textbooks, systematic reviews, meta-analyses, and cross-sectional studies that did not meet the inclusion criteria for study design
- Studies excluded for "Wrong setting" were conducted in populations, geographic locations, or healthcare settings that fell outside the scope of the systematic review's inclusion criteria
- Studies excluded for “Not Retrieved” were due to access issues: Full text not available through institutional subscriptions, Article behind paywall with no open access version, Journal not accessible through available databases.

**PRISMA Excluded Studies Table**

**Excluded Studies (After Full-Text Review)**

| **Study ID** | **Author(s) & Year** | **Title** | **Journal/Source** | **Reason for Exclusion** |
| --- | --- | --- | --- | --- |
| #173 | Carteret (2013) | How culture affects oral health beliefs and behaviors | Dimensions of Culture | Wrong study design |
| #76 | Edberg (2007) | Essentials of health behavior: Social and behavioral theory in public health | Jones & Bartlett Publishers | Wrong study design |
| #170 | Elyasi et al. (2015) | Impact of sense of coherence on oral health behaviors: a systematic review | PLoS One | Wrong setting |
| #171 | Elyasi et al. (2020) | Modeling the Theory of Planned Behaviour to predict adherence to preventive dental visits in preschool children | PLoS One | Wrong setting |
| #77 | Hayden (2022) | Introduction to health behavior theory | Jones & Bartlett Learning | Wrong study design |
| #102 | Lumsden et al. (2021) | Protocol for a family-centered behavioral intervention to reduce early childhood caries: the MySmileBuddy program efficacy trial | BMC Oral Health | Wrong setting |
| #154 | Muller et al. (2024) | Impact of an Oral Health Education Program on the Oral Health Literacy of Refugees | Journal of Immigrant and Minority Health | Wrong setting |
| #103 | Novrinda & Han (2022) | Oral health inequality among Indonesian workers in South Korea: role of health insurance and discrimination factors | BMC Oral Health | Wrong setting |
| #133 | Ntoumanis et al. (2021) | A meta-analysis of self-determination theory-informed intervention studies in the health domain: effects on motivation, health behavior, physical, and psychological health | Health Psychology Review | Wrong study design |
| #157 | Ramos-Gomez & Kinsler (2022) | Addressing social determinants of oral health, structural racism and discrimination and intersectionality among immigrant and non-English speaking Hispanics in the United States | Journal of Public Health Dentistry | Wrong setting |
| #162 | R'Ong et al. (2021) | Oral Health Seeking Behaviour among Non-Malaysian Adults in Malaysia | Medical Journal of Malaysia | Wrong setting |
| #110 | Shi et al. (2021) | Application of the extended theory of planned behavior to understand Chinese students' intention to improve their oral health behaviors: a cross-sectional study | BMC Public Health | Wrong study design |
| #84 | Shmarina et al. (2024) | Oral health literacy among migrant mothers in Sweden. A qualitative study | Acta Odontologica Scandinavica | Wrong setting |
| #111 | Svendsen et al. (2020) | Associations of health literacy with socioeconomic position, health risk behavior, and health status: a large national population-based survey among Danish adults | BMC Public Health | Wrong setting |
| #120 | Tsakos et al. (2023) | Reflections on oral health inequalities: Theories, pathways and next steps for research priorities | Community Dentistry and Oral Epidemiology | Wrong study design |
| #95 | Wang et al. (2022) | Teenager dietary behavior and health literacy in China: influencing factors and coping strategies | Archives of Medical Science | Wrong setting |
| #82 | Wendt et al. (1996) | Analysis of caries-related factors in infants and toddlers living in Sweden | Acta Odontol Scand | Wrong setting |
| - | Lien et al. (2008) | Non-western immigrants' satisfaction with the general practitioners' services in Oslo, Norway | International Journal for Equity in Health | Wrong study design |
| - | Groholt et al. (2008) | Overweight and obesity among adolescents in Norway: cultural and socio-economic differences | Journal of Public Health | Wrong setting |
| - | Sexton & Sorlie (2008) | Use of traditional healing among Sami psychiatric patients in the north of Norway | International Journal of Circumpolar Health | Wrong setting |
| - | Hansen et al. (2015) | Cultural activity participation and associations with self-perceived health, life-satisfaction and mental health: the Young HUNT Study, Norway | BMC Public Health | Wrong setting |
| - | Rabanal et al. (2015) | Ethnic inequalities in acute myocardial infarction and stroke rates in Norway 1994-2009: a nationwide cohort study (CVDNOR) | BMC Public Health | Wrong study design |
| - | Gimeno-Feliu et al. (2016) | Patterns of pharmaceutical use for immigrants to Spain and Norway: a comparative study of prescription databases in two European countries | International Journal for Equity in Health | Wrong setting |
| - | Arora et al. (2019) | Ethnic boundary-making in health care: Experiences of older Pakistani immigrant women in Norway | Social Science & Medicine | Wrong setting |
| - | Straiton et al. (2019) | Perceived discrimination, health and mental health among immigrants in Norway: the role of moderating factors | BMC Public Health | Wrong study design |
| - | Damsgård et al. (2020) | Persistent pain associated with socioeconomic and personal factors in a Sami and Non-Sami population in Norway: an analysis of SAMINOR 2 survey data | International Journal of Circumpolar Health | Wrong setting |
| - | Ohm et al. (2020) | Health care utilisation for treatment of injuries among immigrants in Norway: a nationwide register linkage study | Injury Epidemiology | Wrong setting |
| - | Madar et al. (2022) | Self-reported health and associated factors among the immigrant populations in Norway | Journal of Public Health-Heidelberg | Wrong study design |
| - | Dunlavy et al. (2023) | Health outcomes in young adulthood among former child refugees in Denmark, Norway and Sweden: A cross-country comparative study | Scandinavian Journal of Public Health | Wrong setting |
| - | Kjøllesdal et al. (2023) | Proficiency in the Norwegian language and self-reported health among 12 immigrant groups in Norway: A cross-sectional study | Scandinavian Journal of Public Health | Wrong study design |
| - | Åstrøm (2004) | Stability of oral health‐related behaviour in a Norwegian cohort between the ages of 15 and 23 years | Community Dentistry and Oral Epidemiology | Wrong setting |
| - | Astrøm (2008) | Applicability of action planning and coping planning to dental flossing among Norwegian adults: a confirmatory factor analysis approach | European Journal of Oral Sciences | Wrong setting |
| - | Åstrøm & Wold (2012) | Socio‐behavioural predictors of young adults' self‐reported oral health: 15 years of follow‐up in the The Norwegian Longitudinal Health Behaviour study | Community Dentistry and Oral Epidemiology | Wrong setting |
| - | Shmarina et al. (2023) | Oral health literacy among migrant mothers in Sweden. A qualitative study | Acta Odontologica Scandinavica | Wrong setting |
| - | Tenani et al. (2020) | Influence of oral health literacy on dissatisfaction with oral health among older people | Gerodontology | Wrong setting |
| - | Neves et al. (2020) | The impact of oral health literacy and family cohesion on dental caries in early adolescence | Community Dentistry and Oral Epidemiology | Wrong setting |
| - | Walter et al. (2011) | Oral health‐related quality of life and oral status in a German working population | European Journal of Oral Sciences | Wrong setting |
| - | Bado et al. (2020) | Oral health literacy, self‐rated oral health, and oral health‐related quality of life in Brazilian adults | European Journal of Oral Sciences | Wrong setting |
| - | Lee et al. (2022) | Gender differences in the association between oral health literacy and oral health-related quality of life in older adults | BMC Oral Health | Wrong setting |
| - | Ueno et al. (2013) | Relationship between oral health literacy and oral health behaviors and clinical status in Japanese adults | Journal of Dental Sciences | Wrong setting |
| - | Baskaradoss (2016) | The association between oral health literacy and missed dental appointments | The Journal of the American Dental Association | Wrong setting |
| - | Berkman et al. (2011) | Low health literacy and health outcomes: an updated systematic review | Annals of Internal Medicine | Wrong setting |
| - | Gao & McGrath (2011) | A review on the oral health impacts of acculturation | Journal of Immigrant and Minority Health | Wrong setting |
| - | Fox et al. (2021) | Refugee and migrant health literacy interventions in high-income countries: a systematic review | Journal of Immigrant and Minority Health | Wrong setting |
| - | Manandhar (2014) | Oral health care utilization among immigrants residing in Finland | Itä-Suomen yliopisto | Wrong setting |
| - | Due et al. (2020) | Understanding oral health help‐seeking among Middle Eastern refugees and asylum seekers in Australia: An exploratory study | Community Dentistry and Oral Epidemiology | Wrong setting |
| - | Christensen et al. (2010) | Oral health in children and adolescents with different socio-cultural and socio-economic backgrounds | Acta Odontologica Scandinavica | Wrong setting |
| - | Solyman & Schmidt-Westhausen (2018) | Oral health status among newly arrived refugees in Germany: a cross-sectional study | BMC Oral Health | Wrong setting |
| - | Dahlan et al. (2019) | Impact of acculturation on oral health among immigrants and ethnic minorities: A systematic review | PLoS One | Wrong setting |
| - | Erdsiek et al. (2017) | Oral health behaviour in migrant and non-migrant adults in Germany: the utilization of regular dental check-ups | BMC Oral Health | Wrong setting |
| - | Dahlan et al. (2019) | Impact of social support on oral health among immigrants and ethnic minorities: A systematic review | PLoS One | Wrong setting |
| - | Lauritano et al. (2021) | Oral health status among migrants from middle-and low-income countries to Europe: a systematic review | International Journal of Environmental Research and Public Health | Wrong setting |
| - | Brega et al. (2020) | Health literacy and parental oral health knowledge, beliefs, behavior, and status among parents of American Indian newborns | Journal of Racial and Ethnic Health Disparities | Wrong setting |
| - | Stormacq et al. (2018) | Does health literacy mediate the relationship between socioeconomic status and health disparities? Integrative review | Health Promotion International | Wrong setting |
| - | Schiavo (2011) | Oral health literacy in the dental office: the unrecognized patient risk factor | American Dental Hygienists' Association | Wrong setting |
| - | Lee et al. (2012) | The relationship of oral health literacy and self-efficacy with oral health status and dental neglect | American Journal of Public Health | Wrong setting |
| - | Sukhabogi et al. (2020) | Association of oral health literacy with oral health behavior and oral health outcomes among adult dental patients | Indian Journal of Dental Research | Wrong setting |
| - | Mialhe et al. (2022) | Oral Health Literacy, Sense of Coherence and Associations With Poor School Performance | Journal of School Health | Wrong setting |
| - | Lee (2018) | Lower Oral Health Literacy may lead to Poorer Oral Health Outcomes | Journal of Evidence Based Dental Practice | Wrong setting |
| - | Geltman et al. (2013) | The Impact of Functional Health Literacy and Acculturation on the Oral Health Status of Somali Refugees Living in Massachusetts | American Journal of Public Health | Wrong setting |
| - | Adil et al. (2020) | Assessment of parents' oral health literacy and its association with caries experience of their preschool children | Children | Wrong setting |
| - | Stormacq et al. (2020) | Effects of health literacy interventions on health-related outcomes in socioeconomically disadvantaged adults living in the community: a systematic review | JBI Evidence Synthesis | Wrong setting |
| - | Wu et al. (2021) | Immigration and Oral Health in Older Adults: An Integrative Approach | Journal of Dental Research | Wrong setting |
| - | Weil et al. (2023) | An Evidence-Based Digital Prevention Program to Improve Oral Health Literacy of People With a Migration Background: Intervention Mapping Approach | JMIR Form Res | Wrong setting |
| - | Spinler et al. (2021) | Oral health literacy of persons with migration background—first results of the MuMi study | Bundesgesundheitsblatt-Gesundheitsforschung-Gesundheitsschutz | Wrong setting |

**Summary of Exclusions**

**Total Excluded Studies: 65**

**Studies with documented reasons: 17 (ID-numbered from Covidence)**

- **Wrong study design**: 6 studies (35.3% of documented)
- **Wrong setting**: 11 studies (64.7% of documented)

**Studies needing exclusion reasons: 48**

- **Status**: Excluded studies identified from database searches, End-Mote program, and hand searching.

**Notes**

- First 17 exclusions were made on November 20, 2024, and excluded from Covidence screening.
- For complete PRISMA compliance, the 48 studies marked as excluded (Exclusion categories include: wrong study design, wrong setting, wrong population, wrong intervention, wrong comparator, wrong outcomes).
