# Supplementary material for: The role of oral health literacy in shaping health behaviors among migrants in Norway. An integrative review
Source: BMC Oral Health. 2025 Nov 10;25:1766. doi: 10.1186/s12903-025-07097-6 (PMC12599096; doi:10.1186/s12903-025-07097-6)
Supplement: Supplementary file 3 — Supplementary Material 3 [file 12903_2025_7097_MOESM3_ESM.docx]

| **Section and Topic** | **Item #** | **Checklist item** | **Location where item is reported** |
| --- | --- | --- | --- |
| **TITLE** | | |  |
| Title | 1 | The role of oral health literacy in shaping health behaviors among migrants in Norway. An integrative review | Page 1, Title |
| **ABSTRACT** | | |  |
| Abstract | 2 | **Title**: The role of oral health literacy in shaping health behaviors among migrants in Norway  **Objectives**: A review of the oral health literature to evaluate current evidence and describe oral health literacy and health behavioral changes in the process of migration in Norway  **Eligibility Criteria**: Included peer-reviewed primary research, and both qualitative and quantitative studies conducted in Norway, with a variety of data collection methods and study designs.  **Information Sources**: Databases searched included PubMed, PsycINFO, EBSC, Web of Science, and Google Scholar  **Risk of Bias**: Risk of bias assessed with the CASP Critical Appraisal Skills program and the Mixed Methods Appraisal Tool.  **Results**: Twenty-five peer-reviewed articles involving migrant participants were included, comprising 1 mixed methods study, 18 quantitative and 6 qualitative studies.  **Conclusions**: Some health outcomes are still influenced by the lack of oral health literacy and migrants with low OHL may be more likely to have poor oral health outcomes than the host populations. Emphasizing the need for improvement by understanding migrant health needs for better health services and oral health literacy.  **Registration**: This review was registered with Open Science Framework (OSF) CC-By Attribution 4.0 International. | Page 2, Abstract |
| **INTRODUCTION** | | |  |
| Rationale | 3 | Disparities in oral health represent a global health concern, particularly among migrants. The prevalence of tooth decay and obesity, poor oral health, and limited oral health literacy significantly reduce quality of life. Oral health is often disregarded during acculturation. Identifying the barriers to optimizing the overall health of migrant populations is necessary to address this neglect. | Pages 3-5, Introduction section |
| Objectives | 4 | To assess and identify the indicators of oral health literacy being explored among migrant populations in Norway and OHL associations with health behaviors and health outcomes. An in-depth analysis of the literature is crucial as well as an evaluation of available tools for OHL assessments in clinical settings and primary care. | Pages 4-7, Introduction section |
| **METHODS** | | |  |
| Eligibility criteria | 5 | The inclusion criteria incorporated peer-reviewed primary research, both qualitative and quantitative studies conducted in Norway (a variety of data collection methods and study designs). Studies written in the English language, including open-access publications, and published within the last 20 years. Exclusion criteria included dissertations, editorials, reviews, and secondary sources. Studies written in the Norwegian language and published in Norwegian research journals were also excluded. | Pages 7-8, Methods section |
| Information sources | 6 | The guided search strategy included PubMed, PsycINFO, EBSC, Web of Science, and Google Scholar databases. Last search September 2024. | Pages 8-9, Methods section |
| Search strategy | 7 | Search terms included “oral health literacy,” “health literacy”, “migrants and immigrants”, “health behavior”, and “acculturation or cultural differences”. | Page 9, Methods section |
| Selection process | 8 | A reviewer independently screened titles and abstracts, followed by a full-text review for eligibility. | Page 10-11, Methods section |
| Data collection process | 9 | Data were extracted using a standardized form, including study characteristics, participant demographics, interventions, and outcomes. | Pages 10-11, Methods section |
| Data items | 10a | The following data items from each included study were extracted: author(s), year of publication, study design, sample size, age, gender, inclusion/exclusion criteria, details of the intervention, primary and secondary outcomes, and associations. | Page 11, Methods section |
|  | 10b | Using oral health literacy as the role of the mediator through knowledge, socioeconomic factors (e.g., education and employment), health behavior, other intervention characteristics, and acculturation aspects (e.g. language barriers, system navigation). | Pages 4-5, and 10, Introduction and Methods sections |
| Study risk of bias assessment | 11 | Risk of bias assessment followed the Critical Appraisal Skills Program (CASP) and the Mixed Methods Appraisal Tool (MMAT). The findings of these studies were evaluated in a descriptive manner. | Page 11, Methods section |
| Effect measures | 12 | The primary outcomes were the lack of oral health literacy measures in the studies selected and the lifestyle changes due to the interventional studies | Page 11, Methods section |
| Synthesis methods | 13a | Covidence and End-Note programs were used to extract information about the research questions and the study results relevant to the aims of the integrative review. | Pages 9 & 12, Methods section |
|  | 13b | NVivo program was used to code themes for the conceptual map of outcomes and key themes on OHL. | Page 10 & 12, Methods section |
|  | 13c | The checklists supported the quality of the data extraction | Page 9, Methods section |
|  | 13d | Not applicable. A meta-analysis was not conducted due to the heterogeneity of the study designs, formats, and outcomes. Therefore, no statistical methods were used. | N/A |
|  | 13e | Not applicable. No meta-analysis was performed; therefore, exploring methods for heterogeneity was not relevant. This integrative review was conducted without a formal GRADE assessment. | N/A |
|  | 13f | Not applicable. No quantitative synthesis was conducted, so sensitivity analyses to assess the robustness of results were not performed. | N/A |
| Reporting bias assessment | 14 | CASP and mixed methods appraisal program | Page 11, Methods section |
| Certainty assessment | 15 | Thematic analysis and coding framework | Page 10, Methods section |
| **RESULTS** | | |  |
| Study selection | 16a | Out of 408 records screened, 261 references were removed, 48 full-text articles were assessed for eligibility, and 25 studies were included. | Page 12, Results section |
|  | 16b | Thirty-five studies were not retrieved, 64 were excluded due to not relevant interventions and populations, and studies conducted were not applicable to the review | Page 12, Results section |
| Study characteristics | 17 | Included studies varied in sample size, country of the participant, setting, and delivery method. | Page 13, Results section |
| Risk of bias in studies | 18 | Some studies had a low risk of bias and sufficient detail providing enough information and explaining the method used. | Page 14, Results section |
| Results of individual studies | 19 | The sample size was appropriate. Outcomes related to social and integration aspects were associated with OHL. | Page 12, Results section; Table 2 |
| Results of syntheses | 20a | No studies measured oral health literacy directly in the migrant population with the appropriate tools and instruments. | Page 12, Results sections; Table 2 |
|  | 20b | Not applicable. This is an integrative review without statistical synthesis or meta-analysis. | N/A |
|  | 20c | Studies highlighted the importance of health information, knowledge, and the ability to access healthcare services associated with OHL | Page 14, Results section |
|  | 20d | Not applicable, because no sensitivity analysis was conducted for results assessments. | N/A |
| Reporting biases | 21 | Lack of data from migrants in longer-term studies. Differences in barriers and accessing health services | Pages 14-15, Results section |
| Certainty of evidence | 22 | No inconsistencies, publication bias, and evidence applied to the study population and intervention to some extent. | Pages 14-15, Results section |
| **DISCUSSION** | | |  |
| Discussion | 23a | Key findings indicated the need for improvement, health management, and the impact on health literacy in the selected populations. | Page 15-16, Discussion section |
|  | 23b | Limited focus on oral health literacy and no OHL studies using the appropriate tools in Norway. Validating OHL instruments for the Norwegian and migrant populations is necessary. | Pages 18-19, Discussion section |
|  | 23c | Some studies had small sample sizes and limited data from migrants living in smaller urban and rural areas in Norway | Page 20, Discussion section |
|  | 23d | Focus on OHL, cultural stress theories, and other implications for behavioral health, overcoming the identified challenges to enhance solutions, policy interventions, and long-term outcomes. | Pages 20-21, Discussion section |
| **OTHER INFORMATION** | | |  |
| Registration and protocol | 24a | Review registered in OSF - CC by authorization – Registered in Covidence software platform. | Pages 7 & 12, Methods section |
|  | 24b | The review registration can be accessed in OSF - Protocol was not prepared. | Pages 7 & 12, Methods section |
|  | 24c | Describe and explain any amendments to information provided at registration or in the protocol. | Page 7, Methods section |
| Support | 25 | This review was supported by the Oral Health Centre of Expertise in Western Norway and the COP study | Page 23, Declarations |
| Competing interests | 26 | No competing interests to declare | Page 23, Declarations |
| Availability of data, code and other materials | 27 | Not Applicable. No datasets or supplementary material were shared or made available. | N/A |

*From:*  Page MJ, McKenzie JE, Bossuyt PM, Boutron I, Hoffmann TC, Mulrow CD, et al. The PRISMA 2020 statement: an updated guideline for reporting systematic reviews. BMJ 2021;372:n71. doi: 10.1136/bmj.n71

For more information, visit: <http://www.prisma-statement.org/>
